# Reduced BOLD-CSF coupling as a potential functional imaging correlate of semantic fluency decline in MRI-negative temporal lobe epilepsy

**DOI:** 10.3389/fnagi.2026.1879851

**Published:** 2026-08-19

**Authors:** Ying Xu, Xiaona Xia, Ying Wang, Ke Xue, Ying Wei, Ling Li, Xiangshui Meng

**Affiliations:** 1 Department of Radiology, Qilu Hospital of Shandong University, Jinan, China; 2 Department of Radiology, Qilu Hospital (Qingdao), Cheeloo College of Medicine, Shandong University, Qingdao, China; 3 Medical Imaging and Engineering Intersection Key Laboratory of Qingdao, Qingdao, China; 4 MR Research Collaboration, Shanghai United Imaging Healthcare Co., Ltd., Shanghai, China; 5 Department of Research and Development, Shanghai United Imaging Intelligence Co., Ltd., Shanghai, China; 6 Department of Neurology, Qilu Hospital (Qingdao), Cheeloo College of Medicine, Shandong University, Qingdao, China

**Keywords:** BOLD-CSF coupling, cognitive impairment, glymphatic system, MRI-negative temporal lobe epilepsy, semantic verbal fluency

## Abstract

**Introduction:**

This study investigated neurovascular–cerebrospinal fluid dynamic interactions using blood-oxygen-level-dependent–cerebrospinal fluid (BOLD-CSF) coupling metrics and examined their associations with cognitive impairment in patients with MRI-negative unilateral temporal lobe epilepsy (TLE).

**Methods:**

In this retrospective study, 36 patients with MRI-negative unilateral TLE and 26 healthy controls (HCs) recruited during the same study period were enrolled. Resting-state functional MRI (rs-fMRI) was used to quantify global BOLD-CSF (gBOLD-CSF) and temporal BOLD-CSF (tBOLD-CSF) coupling. Comprehensive neuropsychological assessments included the Montreal Cognitive Assessment (MoCA), Digit Symbol Substitution Test (DSST), Digit Span Test (DST), Block Design, Phonological Fluency Test (PFT), and semantic verbal fluency (SVF). Partial correlations and multivariable linear regression were performed to evaluate associations between imaging metrics and cognitive performance.

**Results:**

Compared with HCs, TLE patients showed significantly poorer performance on MoCA (*p* = 0.007), DSST, DST, Block Design, PFT, and SVF (all *p* < 0.001). Both gBOLD-CSF and tBOLD-CSF coupling strengths were markedly reduced in TLE patients (FDR-*p* < 0.001 for both). After adjustment for age, sex, education level, and sleep duration, both coupling metrics were positively associated with SVF performance. False discovery rate (FDR) correction was applied to the three prespecified primary imaging–SVF associations–gBOLD–CSF coupling, tBOLD–CSF coupling, and CPV–using the Benjamini-Hochberg procedure. The associations between the BOLD–CSF coupling metrics and SVF performance remained significant after FDR correction (gBOLD-CSF coupling: partial *r* = 0.723, unadjusted *p* < 0.001, FDR-adjusted *p* < 0.001; tBOLD-CSF coupling: partial *r* = 0.396, unadjusted *p* = 0.025, FDR-adjusted *p* = 0.038).

**Discussion:**

Reduced tBOLD–CSF and gBOLD–CSF coupling may serve as functional imaging correlates of semantic network vulnerability in patients with MRI-negative TLE.

## 1 Introduction

Epilepsy is a common and disabling neurological disorder affecting more than 70 million people worldwide (Thijs et al., 2019). Epileptic seizures are primarily caused by abnormal, excessive, and synchronous neuronal discharges, which may lead to structural and functional alterations in cortical and subcortical networks over time (Beghi, 2020; Tai et al., 2016). Temporal lobe epilepsy (TLE) is one of the most common forms of focal epilepsy and accounts for approximately 40% of epilepsy cases (Beghi, 2020). Beyond recurrent seizures, TLE is frequently associated with cognitive impairment involving memory, attention, executive function, processing speed, and language-related abilities (Zhao et al., 2014). These cognitive deficits can substantially reduce quality of life and impose a considerable social and clinical burden (Meneses et al., 2009).

Approximately 30% of patients with TLE show no clear epileptogenic lesion on routine structural MRI, a condition commonly referred to as MRI-negative or non-lesional TLE (Muhlhofer et al., 2017). In these patients, epileptogenic localization is often more challenging, and surgical decision-making usually requires additional multimodal evaluation, including long-term electroencephalographic monitoring, functional imaging, and sometimes invasive intracranial recording. Importantly, the absence of visible MRI abnormalities does not necessarily indicate the absence of underlying network dysfunction or progressive cognitive vulnerability. Therefore, identifying sensitive and non-invasive imaging markers associated with early cognitive impairment in MRI-negative TLE is of substantial clinical importance.

In recent years, the glymphatic system (GS), a brain-wide pathway involved in cerebrospinal fluid (CSF)–interstitial fluid exchange and metabolic waste clearance, has attracted increasing attention in neurological disorders (Taoka and Naganawa, 2020). Experimental and clinical studies suggest that altered glymphatic-related physiology may contribute to the accumulation of neurotoxic proteins, neuroinflammation, and cognitive decline. In TLE, reactive astrogliosis, inflammatory activation, and altered aquaporin-4 (AQP4) expression may disturb perivascular fluid transport and compromise glymphatic-related clearance mechanisms (Zou et al., 2024). Human imaging evidence has also suggested that enlarged perivascular spaces and reduced diffusion tensor imaging analysis along the perivascular space index may reflect impaired glymphatic function in patients with TLE (Xie et al., 2024). However, these imaging markers primarily provide structural or relatively static assessments and may not fully capture dynamic interactions between neural activity, hemodynamics, and CSF movement.

Recent advances in resting-state functional MRI (rs-fMRI) have demonstrated that low-frequency global blood-oxygen-level-dependent (BOLD) fluctuations are temporally coupled with CSF signal oscillations, providing a non-invasive functional proxy for neurovascular and CSF dynamic interactions (Kiviniemi et al., 2016; Fultz et al., 2019; Han et al., 2021). In this framework, low-frequency BOLD activity is thought to precede CSF inflow-related signal changes, and the time-lagged BOLD-CSF relationship has been proposed to reflect, at least in part, physiological processes relevant to glymphatic transport. Reduced BOLD-CSF coupling has been reported in several neurological and neurodegenerative conditions and has been associated with pathological protein accumulation and cognitive impairment (Han et al., 2021; Zhu et al., 2025). Nevertheless, BOLD-CSF coupling should be interpreted as an indirect imaging surrogate of glymphatic-related physiology rather than a direct measurement of solute clearance. To date, whether BOLD-CSF coupling is altered in MRI-negative TLE, and whether such alterations are associated with cognitive dysfunction, remains unclear.

Semantic verbal fluency (SVF) is a cognitive function closely related to temporal lobe semantic memory networks (Ralph et al., 2016). Compared with phonological fluency, which relies more heavily on frontal executive search and phonological retrieval, SVF depends more strongly on the integrity of temporal lobe semantic systems, particularly anterior and lateral temporal regions involved in conceptual processing (Ralph et al., 2016; Binney et al., 2010; Henry and Crawford, 2004). Because the temporal lobe is the primary epileptogenic region in TLE, SVF may represent a sensitive cognitive domain for detecting temporal network vulnerability (Metternich et al., 2014; Jaimes-Bautista et al., 2015; Xie et al., 2025). Investigating the relationship between BOLD-CSF coupling and SVF may therefore provide insight into the functional relevance of glymphatic-related physiological disturbance in MRI-negative TLE (Chuang et al., 2025; Lee et al., 2022).

In addition to dynamic BOLD-CSF coupling, we included CPV as an exploratory complementary structural marker. The choroid plexus plays an important role in CSF production, blood-CSF barrier regulation, and neuroimmune signaling, and CP enlargement has been linked to neuroinflammation and impaired CSF/glymphatic function in several neurological disorders (Li et al., 2023; Christensen et al., 2022). However, its role in epilepsy, particularly in MRI-negative TLE, remains insufficiently explored. Combining dynamic BOLD-CSF coupling metrics with structural CP volume (CPV) may provide complementary information regarding glymphatic-related alterations in this population. Therefore, we hypothesized that CPV might provide structural information complementary to functional BOLD-CSF coupling and help determine whether glymphatic-related alterations in MRI-negative TLE are accompanied by measurable choroid plexus enlargement.

In this study, we aimed to determine whether BOLD-CSF coupling is altered in patients with MRI-negative unilateral TLE and whether such alterations are associated with domain-specific cognitive impairment. Given the temporal lobe origin of TLE and the role of temporal networks in semantic processing, we examined both global BOLD-CSF coupling and temporal lobe-specific BOLD-CSF (tBOLD-CSF) coupling. We further explored whether CPV, a structural marker potentially related to CSF production and altered glymphatic-related physiology, differs between patients with TLE and healthy controls (HCs). We hypothesized that patients with MRI-negative TLE would show reduced BOLD-CSF coupling and that lower coupling strength would be associated with poorer cognitive performance, particularly in SVF.

## 2 Materials and methods

This study was conducted in accordance with the ethical principles outlined in the Declaration of Helsinki. The study protocol was approved by the Ethics Committee of Qilu Hospital (Qingdao), Shandong University (Approval No. KYLL-qdql.2020070). Informed consent was waived for this retrospective study because of the retrospective nature of the study and anonymized data analysis.

### 2.1 Participants

We retrospectively enrolled patients with unilateral MRI-negative TLE who were consecutively hospitalized in the Department of Neurology at our institution between November 2019 and November 2021. The diagnosis of unilateral TLE was established by epilepsy specialists based on seizure semiology, video-electroencephalographic monitoring, and neuroimaging findings. MRI-negative status was defined as the absence of visible epileptogenic lesions on routine structural MRI, including three-dimensional T1-weighted (3D-T1WI) imaging, T2-weighted imaging or fluid-attenuated inversion recovery imaging, and diffusion-weighted imaging, as reviewed by experienced neuroradiologists (Czarnetzki et al., 2023).

The inclusion criteria were as follows: (1) a clinical diagnosis of unilateral TLE; (2) availability of brain MRI data, including rs-fMRI and high-resolution three-dimensional T1-weighted imaging; and (3) completion of neuropsychological assessment within one week before MRI scanning. All patients were free of clinical seizures within 48 hours before neuropsychological assessment. The exclusion criteria were as follows: (1) any MRI-visible brain parenchymal lesion, including tumor, vascular malformation, traumatic lesion, cortical malformation, or hippocampal sclerosis visible on routine MRI; (2) sleep deprivation within one week before MRI scanning; (3) poor image quality or excessive head motion; and (4) a history of other neurological, psychiatric, or neurodegenerative disorders. None of the patients included in the present cohort underwent surgical treatment during the study period.

**FIGURE 1:**
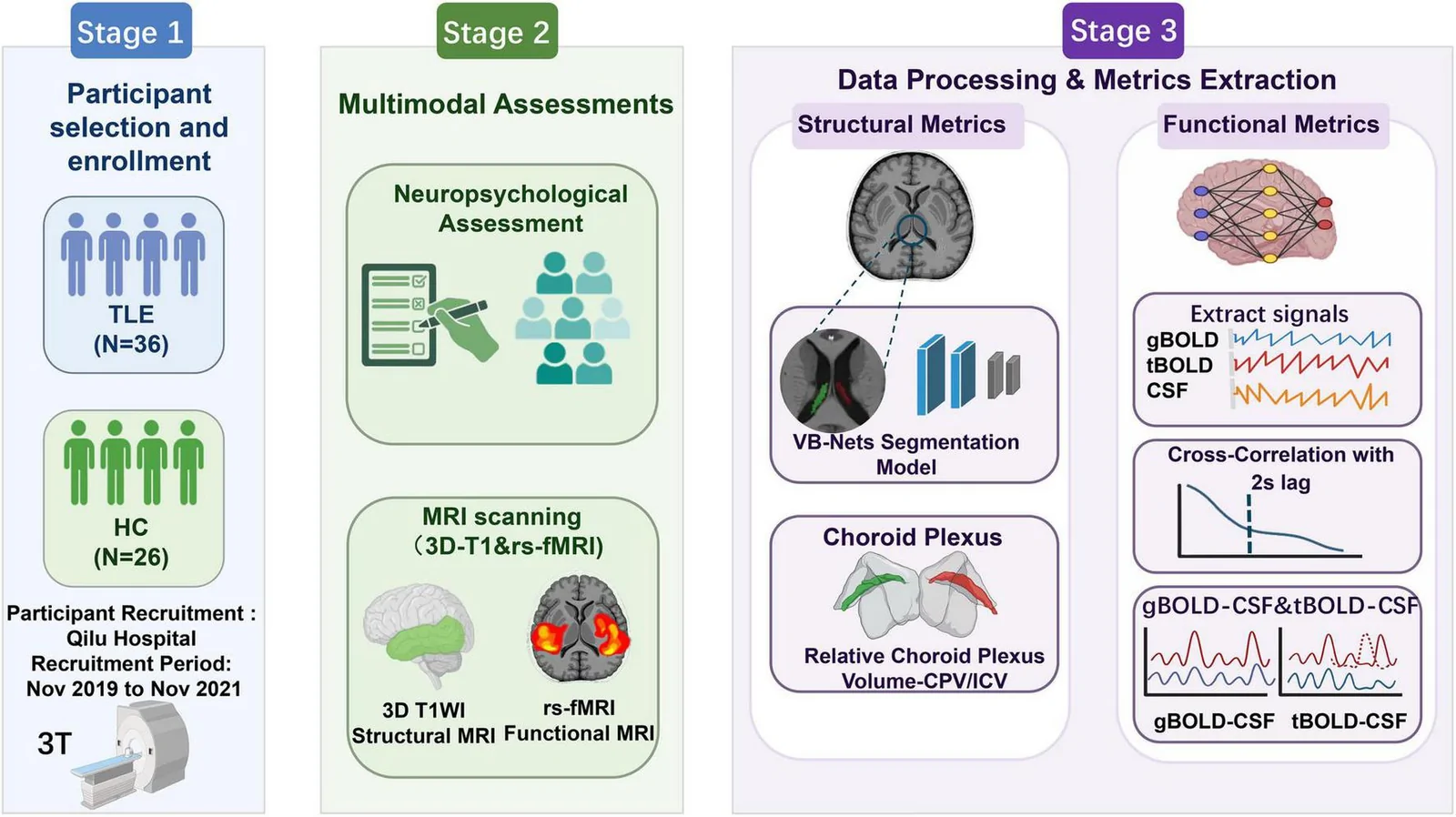
Schematic overview of the study workflow. Stage 1: study design and participant recruitment. Stage 2: multimodal assessments. Stage 3: data processing and metric extraction. TLE, temporal lobe epilepsy; CPV, choroid plexus volume; ICV, intracranial volume; gBOLD, global blood-oxygen-level-dependent; tBOLD, temporal blood-oxygen-level-dependent; CSF, cerebrospinal fluid.

Healthy controls were recruited during the same study period. No individual matching procedure was applied. HCs had no history of neurological or psychiatric disease and showed normal neurological examination and MRI findings. The same exclusion criteria were applied to HCs (Figure 1).

### 2.2 Clinical and cognitive assessments

Baseline clinical data included age, sex, education level, age at seizure onset, disease duration, seizure frequency, average daily sleep duration, Pittsburgh Sleep Quality Index (PSQI) score, and antiseizure medication status. Cognitive function was assessed using a standardized neuropsychological battery, including the Montreal Cognitive Assessment (MoCA), Digit Symbol Substitution Test (DSST), Digit Span Test (DST), Boston Naming Test (BNT), Block Design Test, Phonological Fluency Test (PFT), and SVF. SVF was considered the primary cognitive measure of interest because of its close relationship with temporal lobe semantic memory networks (Figure 1).

### 2.3 MRI acquisition

All participants underwent MRI scanning using the same protocol on a 3T MRI scanner (Ingenia, Philips Healthcare, Netherlands) equipped with an eight-channel phased-array head coil. Routine clinical MRI sequences, including T2-weighted imaging, fluid-attenuated inversion recovery imaging, and diffusion-weighted imaging, were used to exclude visible structural lesions. Quantitative analyses in the present study were based on high-resolution 3D-T1WI imaging and rs-fMRI. To ensure consistency of the acquired imaging data, no major hardware or software upgrades were performed on the MRI system during the study period. During rs-fMRI scanning, participants were instructed to keep their eyes closed, remain awake, minimize head motion, and avoid engaging in any specific mental activity. 3D-T1WI images were acquired using a fast spoiled gradient echo sequence: repetition time (TR) = 6.7 ms, echo time (TE) = 3.0 ms, flip angle (FA) = 8°, field of view (FOV) = 240 mm × 240 mm, data matrix = 240 × 240, slice thickness (ST) = 1 mm, inter-slice gap = 0 mm, number of slices = 170, number of signal averages (NSA) = 1, acquisition orientation = sagittal. Rs-fMRI images were collected using an echo-planar imaging sequence with the following parameters: TR = 2,000 ms, TE = 30 ms, FA = 90°, FOV = 230 mm × 230 mm, data matrix = 68 × 66, ST = 4 mm, gap = 0.5 mm, number of slices = 32, 240 time points, NSA = 1, orientation = transverse (Guan et al., 2021).

Brain region volume calculation: Structural 3D-T1WI images were processed using the uRP platform (Shanghai United Imaging Intelligence Co., Ltd.), which includes skull stripping, bias field correction, and resampling to 1 mm isotropic resolution (Wu et al., 2023). In the present study, the CPV in the bilateral lateral ventricles and intracranial volume (ICV) were quantified using a pre-trained cascaded VB-Nets model integrated in the uRP tool, according to the Desikan-Killiany atlas. Relative CPV (CPV/ICV) was used for subsequent analyses to account for individual differences in head size (Wu et al., 2023). The segmentation accuracy was validated with an average Dice similarity coefficient of 91.06% compared to FreeSurfer (Jeong et al., 2023; Figure 1).

### 2.4 Extraction of global BOLD, temporal BOLD, and CSF signals

**FIGURE 2:**
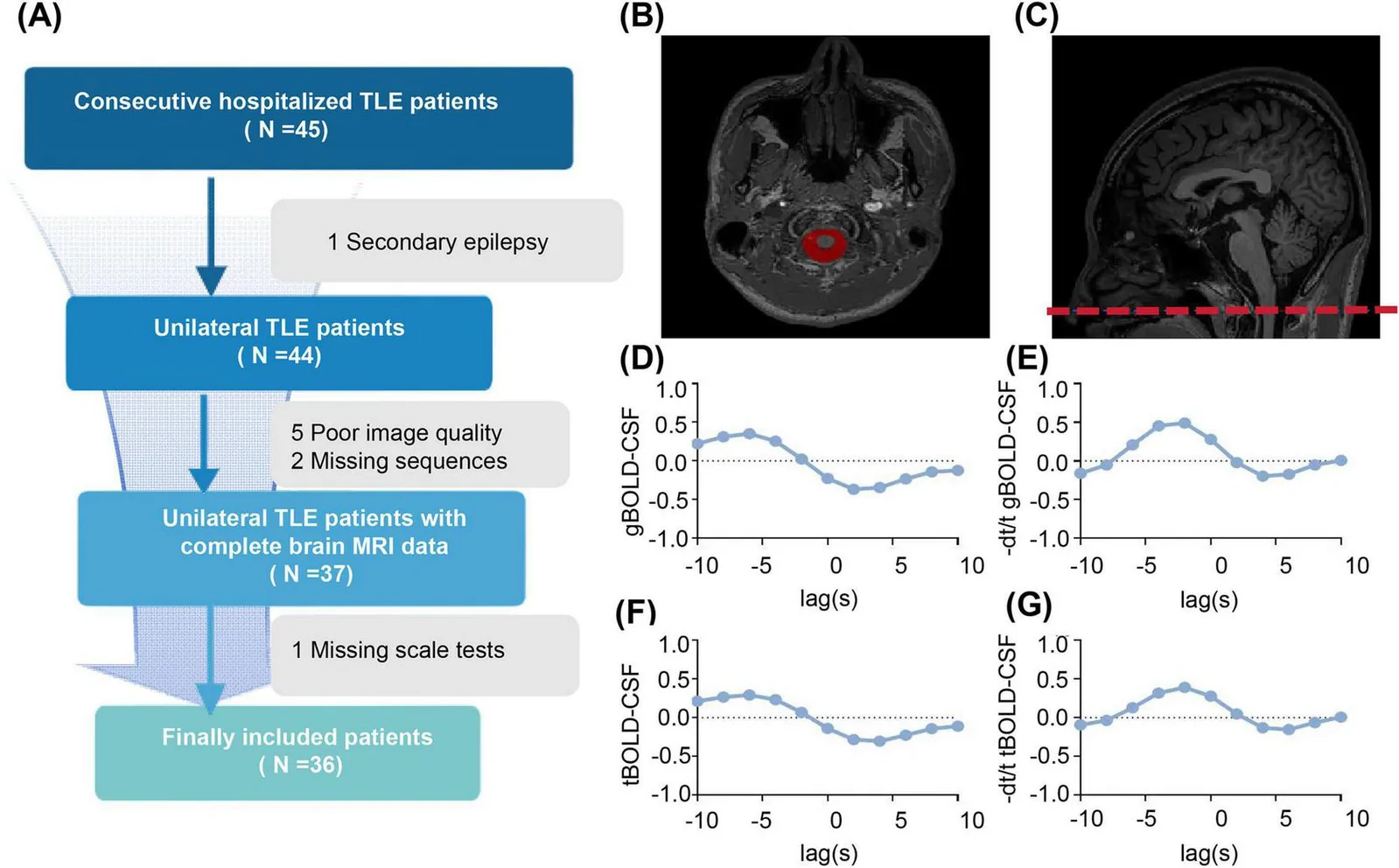
Patient selection flowchart and representative blood-oxygen-level-dependent–cerebrospinal fluid (BOLD-CSF) coupling analysis in unilateral temporal lobe epilepsy (TLE). **(A)** Patient selection flowchart. **(B,C)** Representative cerebrospinal fluid (CSF) signal extraction from the inferior portion of the resting-state functional MRI (rs-fMRI) acquisition volume. **(D,E)** Representative time-lagged cross-correlation analysis between the CSF signal and the global BOLD signal. **(F,G)** Representative time-lagged cross-correlation analysis between the CSF signal and the temporal BOLD signal.

Resting-state functional MRI data were preprocessed using a standard pipeline that included realignment for head-motion correction, slice-timing correction, spatial normalization to Montreal Neurological Institute space, linear detrending, band-pass filtering at 0.01–0.1 Hz, and voxel-wise Z-score normalization. The global BOLD signal was obtained by averaging the preprocessed time series across the cortical gray matter mask derived from the Harvard-Oxford atlas. The temporal BOLD signal was extracted from the bilateral temporal lobe cortical gray matter mask. The CSF signal was extracted from voxels located in the inferior portion of the fMRI acquisition volume, where CSF inflow-related signal changes are most prominent. To reduce contamination from cortical BOLD activity, the CSF region was selected outside cortical gray matter and visually inspected when necessary (Figure 2).

### 2.5 Quantification of BOLD-CSF coupling

Coupling strength was quantified as the cross-correlation coefficient between the BOLD time series and the CSF signal. BOLD-CSF coupling strength was defined as the absolute magnitude of the negative cross-correlation coefficient at a 2s lag for each subject (Han et al., 2021). To be consistent with the gBOLD-CSF coupling, we quantified the tBOLD-CSF coupling at a lag of 2s (Figure 2).

### 2.6 Statistical analyses

All statistical analyses were performed using SPSS (version 27, IBM Corp.) and GraphPad Prism (version 10). Data normality was assessed with the Kolmogorov-Smirnov test. Continuous variables are presented as mean ± standard deviation or median (interquartile range), and categorical variables as counts (percentages). Group differences in continuous variables were examined using independent-samples *t*-tests or Mann-Whitney U tests, as appropriate, and categorical variables with chi-square tests. ANCOVA was used to compare gBOLD-CSF and tBOLD-CSF coupling between groups, with age, sex, education level, and sleep duration as covariates. Partial correlation analyses, adjusted for age, sex, education level, and sleep duration, were performed to examine relationships between imaging metrics, including gBOLD-CSF coupling, tBOLD-CSF coupling, and CPV, and cognitive scores, with false discovery rate correction using the Benjamini-Hochberg procedure (q = 0.05). Multivariable linear regression analysis was performed to identify independent predictors of neuropsychological performance. Variance inflation factors were calculated to assess multicollinearity. In addition, exploratory subgroup analyses were performed to compare cognitive performance and BOLD-CSF coupling metrics between drug-naive and antiseizure medication-treated patients. Disease duration and seizure frequency were additionally examined as continuous clinical variables in exploratory regression analyses. These variables were not dichotomized because arbitrary categorization would reduce statistical power. Given the modest subgroup sizes, exploratory subgroup analyses according to seizure lateralization were also performed to compare gBOLD-CSF coupling, tBOLD-CSF coupling, and SVF performance between patients with left-sided and right-sided TLE, and to examine lateralization-specific associations between BOLD-CSF coupling metrics and SVF performance. Formal stratified analyses according to language dominance were not performed because language-dominance data were not systematically available. A two-tailed *p*-value < 0.05 was considered statistically significant.

## 3 Results

### 3.1 Baseline characteristics of the study participants

The TLE group had a numerically higher median age than the HC group, although the between-group difference did not reach statistical significance (32.0 [21.0–52.8] vs. 23.0 [21.8–34.0] years, *p* = 0.287). No statistically significant differences were observed in sex distribution, education level, average daily sleep duration, or PSQI score (all *p* > 0.05). Compared with HCs, patients with TLE showed significantly poorer performance on MoCA (*p* = 0.007), DSST, DST, Block Design, PFT, and SVF (all *p* < 0.001). No significant between-group difference was observed in BNT performance (*p* = 0.269). Detailed demographic, clinical, and neuropsychological characteristics are summarized in Table 1.

**TABLE 1:** Clinical and neuropsychological characteristics in patients with TLE and HCs.

Clinical characteristics	TLE (*N* = 36)	HCs (*N* = 26)	*P*-value
Age (years)	32.0 (21.0–52.8)	23.0 (21.8–34.0)	0.287
Male, *n* (%)	18 (50.0%)	15 (57.7%)	0.549
Education level	–	–	0.765
Less than college	18 (50.0%)	14 (53.8%)	
College or equivalent	18 (50.0%)	12 (46.2%)	
Daily sleep duration (hours)	8.0 (6.3-8.0)	7.0 (7.0-8.0)	0.249
PSQI	3.0 (2.0-6.0)	4.0 (2.0-5.3)	0.692
Age of first onset of seizure		NA	
<18 years	9 (25.0%)		
≥18 years	27 (75.0%)		
Disease duration of epilepsy (months)	36.0 (12.0-62.8)	NA	
Seizure lateralization			
Left TLE	19 (52.8%)	NA	
Right TLE	17 (47.2%)	NA	
Neuropsychological assessments			
MoCA	27.0 (24–29.0)	29.0 (28.5–30.0)	0.007
DSST	57.0 (42.5-66.0)	72.0 (66.0–75.0)	<0.001
DST	4.0 (3.0-7.0)	8.0 (7.0–8.0)	<0.001
BNT	26.0 (22.5-29.0)	27.0 (26.5–28.0)	0.269
Block design	33.0 (26.5-41.5)	45.0 (42.5–47.5)	<0.001
PFT	8.88 ± 5.8	20.84 ± 6.0	<0.001
SVF	13.4 ± 4.2	17.9 ± 5.1	<0.001

*P* < 0.05 indicates statistically significant. TLE, temporal lobe epilepsy; HCs, healthy controls; PSQI, Pittsburgh Sleep Quality Index; MoCA, Montreal Cognitive Assessment; DSST, Digit Symbol Substitution Test; DST, Digit Span Test; PFT, Phonological Fluency Test; SVF, semantic verbal fluency; NA, not available.

### 3.2 Analysis of BOLD-CSF coupling metrics and CPV

**FIGURE 3:**
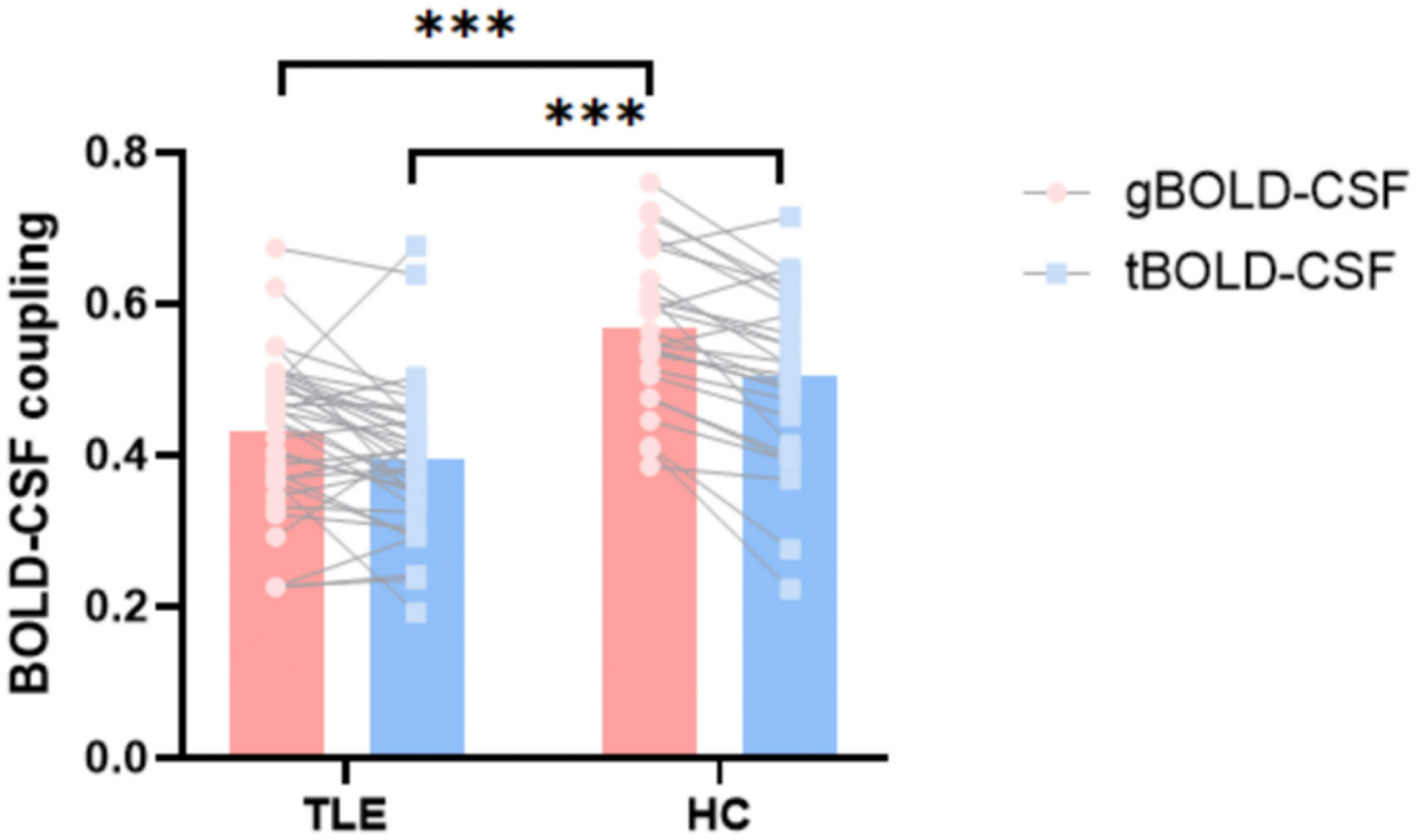
Comparison of global blood-oxygen-level-dependent– cerebrospinal fluid (gBOLD-CSF) and temporal blood-oxygen-level-dependent–cerebrospinal fluid (tBOLD-CSF) coupling between patients with temporal lobe epilepsy (TLE) and healthy controls (HCs). Group differences were assessed using analysis of covariance adjusted for age, sex, education level, and sleep-related variables. ***p < 0.001.

After adjustment for age, sex, education level, and sleep duration, both gBOLD-CSF and tBOLD-CSF coupling were significantly lower in patients with TLE than in HCs (both *p* < 0.001). Within each group, tBOLD-CSF coupling was weaker than gBOLD-CSF coupling (both *p* < 0.001). When the relative reduction in coupling strength was compared between metrics, the reduction in gBOLD-CSF coupling appeared more pronounced than that in tBOLD-CSF coupling (Figure 3). In contrast, relative CPV did not differ significantly between patients with TLE and HCs after adjustment for the same covariates (*p* > 0.05).

### 3.3 Association between BOLD-CSF coupling and cognitive performance

**FIGURE 4:**
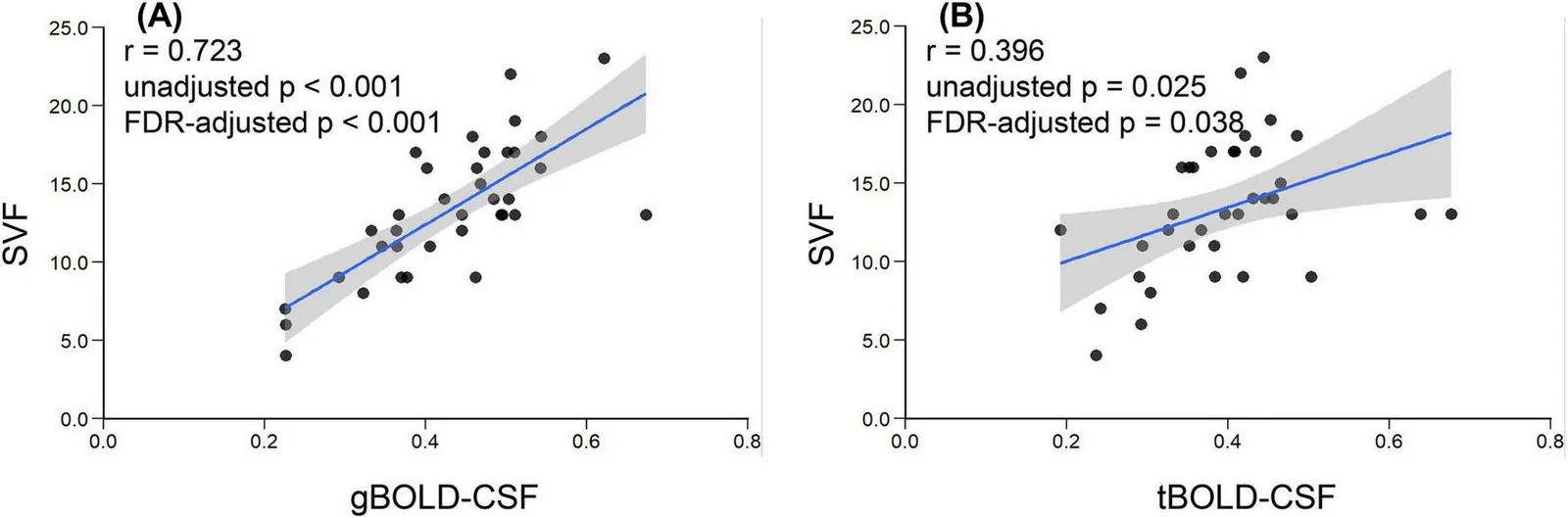
Associations between blood-oxygen-level-dependent–cerebrospinal fluid (BOLD–CSF) coupling metrics and semantic verbal fluency (SVF) performance in patients with unilateral temporal lobe epilepsy (TLE). **(A)** Partial correlation between global blood-oxygen-level-dependent–cerebrospinal fluid (gBOLD–CSF) coupling and SVF score. **(B)** Partial correlation between temporal blood-oxygen-level-dependent–cerebrospinal fluid (tBOLD–CSF) coupling and SVF score. Partial correlation analyses were adjusted for age, sex, education level, and sleep duration. False discovery rate (FDR) correction was applied to the three prespecified primary imaging–SVF associations—gBOLD–CSF coupling, tBOLD–CSF coupling, and CPV—using the Benjamini–Hochberg procedure. Both unadjusted and FDR-adjusted *p*-values are reported (*N* = 36; df = 30).

After controlling for age, sex, sleep duration, and education level, only SVF performance was significantly associated with both gBOLD-CSF and tBOLD-CSF coupling strengths (both FDR-*p* < 0.05); no other cognitive domains showed a significant relationship with either metric (Figure 4). No significant correlation was observed between CPV and any of the cognitive domain scales.

### 3.4 Independent predictors of SVF

Multivariable linear regression analysis showed that lower gBOLD-CSF coupling was independently associated with poorer SVF performance after adjustment for age, sex, education level and sleep duration (β = 0.689, *p* < 0.001; Table 2). GBOLD-CSF coupling substantially improved model fit, accounting for an additional 42.7% of the variance in SVF performance (ΔR^2^; = 0.427, *p* < 0.001). The resulting model was significant overall (R^2^; = 0.609, adjusted R^2^; = 0.544, *p* < 0.001). In a separate adjusted model, higher tBOLD-CSF coupling was also positively associated with SVF performance (β = 0.364, *p* = 0.025). The addition of tBOLD-CSF coupling explained an additional 12.8% of the variance (ΔR^2^; = 0.128, *p* = 0.025), and the overall model was significant (R^2^; = 0.310, adjusted R^2^; = 0.195, *p* = 0.040). In contrast, total choroid plexus volume was not significantly associated with SVF performance after adjustment for the same covariates (β = −0.310, *p* = 0.163), and its addition did not significantly improve model fit (ΔR^2^; = 0.052, *p* = 0.163). All variance inflation factors were below 2, indicating no substantial multicollinearity. *Post hoc* incremental power analysis showed that the addition of gBOLD-CSF coupling yielded an incremental effect size of Cohen’s f^2^; = 1.093 and an achieved statistical power of 1−β = 1.000 at α = 0.05. For tBOLD-CSF coupling, the incremental effect size was f^2^; = 0.186, with an achieved power of 0.655. For total choroid plexus volume, the incremental effect size was f^2^; = 0.068, with an achieved power of 0.299. Given the modest sample size and the limited statistical power for the tBOLD-CSF and choroid plexus volume analyses, these findings should be considered exploratory and require validation in larger independent cohorts (Table 2).

**TABLE 2:** Multivariable linear regression analysis of factors associated with SVF performance in patients with TLE.

Model	Predictor	B	β	*P*-value	Adjusted R^2^
Model 1					0.077
	Age	−0.079	−0.333	0.071	–
	Sex	0.675	0.159	0.631	–
	Sleep duration	−0.559	−0.181	0.297	–
	Education	1.540	0.184	0.297	–
Model 2					0.544
	Age	−0.038	−0.158	0.230	–
	Sex	0.855	0.201	0.390	–
	Sleep duration	−0.056	−0.018	0.884	–
	Education	1.060	0.126	0.310	–
	gBOLD-CSF	28.429	0.689	<0.001	–
Model 3					0.195
	Age	−0.067	−0.280	0.105	–
	Sex	0.669	0.158	0.611	–
	Sleep duration	−0.388	−0.126	0.441	–
	Education	1.740	0.208	0.209	–
	tBOLD-CSF	15.784	0.364	0.025	–
Model 4					0.107
	Age	−0.032	−0.134	0.553	–
	Sex	0.636	0.150	0.646	–
	Sleep duration	−0.228	−0.074	0.690	–
	Education	1.259	0.150	0.389	–
	CPV	−52.889	−0.310	0.163	–

All models included 36 patients with TLE (*N* = 36). Model 1 included age, sex, sleep duration, and education level, with an overall model test of F (4, 31). Models 2–4 separately added gBOLD–CSF coupling, tBOLD–CSF coupling, or CPV to the same four covariates, with an overall model test of F(5, 30). SVF, semantic verbal fluency test; gBOLD, global blood-oxygen-level-dependent; tBOLD, temporal blood-oxygen-level-dependent; CSF, cerebrospinal fluid; CPV, choroid plexus volume.

### 3.5 Association of antiseizure medications, cognitive function, and BOLD-CSF coupling in TLE

Exploratory subgroup analyses were performed to compare cognitive performance and BOLD-CSF coupling between drug-naive patients and patients receiving ASMs. Mann-Whitney U tests showed no statistically significant differences in cognitive scores or BOLD-CSF coupling metrics between the two subgroups (all *p* > 0.05). However, because only six patients were drug-naive, these analyses were underpowered, and potential effects of ASMs on neurovascular activity, cerebral perfusion, and BOLD-CSF coupling cannot be excluded.

### 3.6 Exploratory lateralization analyses

To further address the potential influence of seizure lateralization, patients with TLE were stratified into left-sided and right-sided TLE subgroups. There were 19 patients with left-sided TLE and 17 patients with right-sided TLE. No significant between-subgroup difference was observed in gBOLD-CSF coupling strength between the left and right TLE groups (0.422 ± 0.105 vs. 0.442 ± 0.103, FDR-corrected *p* = 0.709). Similarly, tBOLD-CSF coupling did not differ significantly between the two subgroups (0.366 ± 0.085 vs. 0.427 ± 0.104, FDR-corrected *p* = 0.549). SVF performance was also comparable between the left and right TLE groups (13.1 ± 4.18 vs. 13.6 ± 4.43, FDR-corrected *p* = 0.709). None of these between-subgroup differences remained significant after correction for multiple comparisons.

Exploratory partial correlation analyses were subsequently performed within each lateralization subgroup, controlling for age and sex. Neither gBOLD-CSF nor tBOLD-CSF coupling was significantly associated with SVF performance in either the left or right TLE subgroup. These exploratory findings did not demonstrate a statistically significant lateralization-specific association between BOLD-CSF coupling and SVF performance, although the analyses were limited by the modest subgroup sample sizes.

## 4 Discussion

This study demonstrated that patients with MRI-negative unilateral TLE exhibited significantly reduced gBOLD-CSF and tBOLD-CSF coupling compared with HCs. These coupling alterations were associated with impaired SVF performance. In separate models adjusted for demographic and sleep-related covariates, lower gBOLD–CSF coupling and lower tBOLD–CSF coupling were each associated with poorer SVF performance. These findings demonstrate an association between altered BOLD-CSF coupling and domain-specific cognitive impairment in MRI-negative TLE. Although BOLD-CSF coupling may reflect glymphatic-related physiology, it should be interpreted as an indirect functional imaging correlate rather than a direct measure of glymphatic solute clearance.

An important finding of this study was that gBOLD-CSF coupling showed stronger independent association with SVF performance than tBOLD-CSF coupling. Although SVF is closely related to temporal lobe semantic networks, TLE is increasingly recognized as a network disorder rather than a strictly focal disease (Aghakhani et al., 2015). Interictal and ictal activity may propagate through distributed temporal, limbic, thalamic, and cortical circuits, leading to widespread alterations in neurovascular and CSF dynamic interactions (Aghakhani et al., 2015; Salimeen et al., 2021; Verheggen et al., 2018). Recurrent interictal discharges and altered neurovascular regulation may influence physiological processes contributing to CSF dynamics and glymphatic-related transport. Therefore, gBOLD-CSF coupling may capture the cumulative burden of network-level physiological disruption more effectively than a temporal lobe-restricted metric. Another possibility is that tBOLD-CSF coupling based on a predefined bilateral temporal mask may not fully reflect the heterogeneous lateralization and spatial extent of epileptogenic networks in individual patients (Han et al., 2021; Avesani et al., 2014; Farhan et al., 2025; Iliff et al., 2012; Wang et al., 2023; Toral-Rios et al., 2020; Fultz et al., 2019). These findings suggest that global BOLD-CSF coupling may be a more sensitive marker of cognitive vulnerability in MRI-negative TLE, although this interpretation requires confirmation in larger studies with individualized epileptogenic network mapping.

Notably, reduced BOLD-CSF coupling in our study was selectively associated with SVF impairment, whereas weaker or no associations were observed with PFT and other cognitive domains. The selective association between BOLD-CSF coupling and SVF impairment is biologically plausible. Although both SVF and PFT require executive control and lexical retrieval, they rely on partially distinct cognitive and neural systems. PFT depends more strongly on frontal executive search strategies and phonological processing, whereas SVF is more dependent on temporal lobe semantic memory systems, particularly anterior and lateral temporal regions that support conceptual knowledge (Metternich et al., 2014; Landin-Romero et al., 2016). Because the temporal lobe is the primary pathological substrate in TLE, semantic networks may be particularly vulnerable to epilepsy-related physiological disruption (Blümcke et al., 2013; Grogan et al., 2009). Reduced BOLD-CSF coupling may therefore be more closely related to impairment in temporal lobe-dependent semantic processing than to generalized cognitive decline. This interpretation is consistent with the observation that BOLD-CSF coupling was associated with SVF but not with all cognitive domains examined in the present study.

The selective relationship between reduced BOLD-CSF coupling and SVF performance should be interpreted with caution, given the clinical heterogeneity of patients with TLE. SVF depends on temporal semantic networks and may be influenced by seizure lateralization, language-hemisphere dominance, seizure burden, and disease duration. To further address the potential influence of seizure lateralization, we performed exploratory analyses stratified by left-sided and right-sided TLE. No significant differences were observed between the two subgroups in gBOLD-CSF coupling, tBOLD-CSF coupling, or SVF performance, and these findings remained non-significant after correction for multiple comparisons. Similarly, after correction for multiple comparisons, within-subgroup partial correlation analyses controlling for age and sex showed no significant associations between either gBOLD-CSF or tBOLD-CSF coupling and SVF performance in the left or right TLE subgroup.

These findings should be interpreted cautiously because the left and right TLE subgroups were small, limiting the statistical power to detect lateralization-dependent effects. In addition, formal language-dominance assessment was not systematically available; therefore, left-sided seizure lateralization could not be directly equated with involvement of the language-dominant hemisphere. Moreover, tBOLD-CSF coupling was calculated using a bilateral temporal lobe mask, which may have averaged distinct ipsilateral and contralateral effects. Although disease duration and seizure frequency were not significantly associated with BOLD-CSF coupling, these negative results do not exclude clinically relevant modifying effects given the modest sample size and variability in cumulative seizure burden. Therefore, our findings should be regarded as cohort-level evidence linking altered BOLD-CSF coupling to semantic fluency impairment, rather than evidence for a lateralization-specific mechanism.

Although our cohort had a median disease duration of 36.0 months, significant BOLD-CSF coupling reduction was observed in the absence of remarkable CPV group differences and cognitive correlates of CPV. CPV was included as an exploratory complementary structural marker because the choroid plexus is involved in CSF production, immune signaling, and blood–CSF barrier regulation (Tireli et al., 2025). We initially hypothesized that CPV might provide structural evidence related to altered glymphatic-related physiology in MRI-negative TLE. However, relative CPV did not differ between patients and HCs and was not associated with cognitive performance. This negative finding suggests that altered BOLD-CSF coupling in MRI-negative TLE may occur without detectable choroid plexus enlargement. One possible explanation is that BOLD-CSF coupling reflects dynamic interactions among neural activity, hemodynamic fluctuations, and CSF motion, whereas CPV is a relatively coarse structural measure (Fultz et al., 2019). In MRI-negative TLE, recurrent epileptic activity, neurovascular dysregulation, and subtle perivascular or astroglial alterations may disrupt CSF-related dynamics before overt structural changes of the choroid plexus become measurable (Yin et al., 2022; Lee et al., 2022; Rasmussen et al., 2018). Choroid plexus enlargement may instead reflect more chronic or severe neuroinflammatory processes, blood–CSF barrier disruption, or longer disease burden (Tireli et al., 2025). Therefore, the dissociation between reduced BOLD-CSF coupling and unchanged CPV may indicate that functional BOLD-CSF coupling is a more sensitive marker of early glymphatic-related disturbance than CPV in this population (Kuang et al., 2026; Fleischer et al., 2026). Nevertheless, because CPV was assessed cross-sectionally, longitudinal studies are needed to determine whether CP structural changes emerge with disease progression or greater inflammatory burden.

Antiseizure medication (ASM) use represents an important confounding factor in neuroimaging studies of epilepsy, as most ASMs modulate neuronal excitability, cerebral blood flow, and neural activity reflected by BOLD signals (Kim et al., 2023). Several ASMs, including oxcarbazepine, lamotrigine, valproate, levetiracetam, and topiramate, have been reported to alter cerebral perfusion, neural oscillatory activity, and potentially glymphatic-related fluid dynamics, which may in turn affect BOLD-CSF coupling (Kim et al., 2023; Iasinovschi et al., 2026; Elabasy et al., 2023). In the present study, only six patients were drug-naive, while the remaining 30 were receiving various ASM regimens, including monotherapy and polytherapy. Although exploratory subgroup comparison did not reveal statistically significant differences in BOLD-CSF coupling between drug-naive and ASM-treated patients, likely due to the small sample size of the drug-naive subgroup, we cannot fully exclude potential modulatory effects of ASMs on the observed reductions in BOLD-CSF coupling. Future studies with larger samples of drug-naive patients are warranted to clarify the specific contribution of epilepsy itself independent of ASMs.

Several limitations should be acknowledged. First, the sample size was modest, particularly for multivariable regression analyses, subgroup comparisons according to antiseizure medication status, and exploratory analyses stratified by seizure lateralization. Therefore, the present findings should be considered exploratory and require validation in larger independent cohorts. Second, the retrospective and cross-sectional design precludes causal inference. Although reduced BOLD-CSF coupling was associated with poorer semantic verbal fluency performance, this relationship should be interpreted as an association rather than evidence of direct causal dependence. It remains unclear whether altered BOLD-CSF coupling contributes to cognitive decline, occurs as a consequence of recurrent seizures, neurovascular dysregulation, inflammation, or medication exposure, or simply coexists with semantic network dysfunction in MRI-negative TLE. Third, postoperative and histopathological data were not available in the present cohort because none of the enrolled patients underwent surgical treatment during the study period. Therefore, we were unable to examine the relationship between BOLD-CSF coupling and surgical outcomes, tissue pathology, postoperative imaging changes, or longitudinal postoperative cognitive trajectories. Future prospective studies including surgically treated patients with MRI-negative TLE are needed to determine whether BOLD-CSF coupling has prognostic value or pathological relevance. Fourth, although sleep duration and PSQI score were included as covariates, objective sleep architecture was not assessed. Because slow-wave sleep is closely related to glymphatic activity, future studies incorporating polysomnography are warranted. Fifth, physiological signals such as respiration and cardiac activity were not recorded during rs-fMRI, and these factors may influence both BOLD and CSF signal fluctuations. Sixth, BOLD-CSF coupling is an indirect functional imaging proxy of neurovascular-CSF dynamic interactions and should not be interpreted as a direct measurement of glymphatic solute clearance.

## 5 Conclusion

In conclusion, patients with MRI-negative unilateral TLE exhibited reduced gBOLD-CSF and tBOLD-CSF coupling. In separate adjusted models, lower gBOLD-CSF coupling and lower tBOLD-CSF coupling were each selectively associated with poorer SVF performance. Of the two separate models, the model including gBOLD-CSF coupling showed the stronger association with SVF. These findings suggest that altered BOLD-CSF coupling may represent a functional imaging correlate of disrupted neurovascular–CSF dynamics and semantic network vulnerability in MRI-negative TLE.

## Data Availability

The raw data supporting the conclusions of this article will be made available by the authors, without undue reservation.
